# Innovative approaches to vector control: integrating genomic, biological, and chemical strategies

**DOI:** 10.1097/MS9.0000000000003469

**Published:** 2025-06-10

**Authors:** Ebrahim Abbasi

**Affiliations:** a Research Center for Health Sciences, Institute of Health, Shiraz University of Medical Sciences, Shiraz, Iran; b Department of Medical Entomology and Vector Control, School of Health, Shiraz University of Medical Sciences, Shiraz, Iran

**Keywords:** biological interventions, chemical innovations, CRISPR/Cas9, genomic strategies, integrated vector management, resistance, vector-borne diseases, Wolbachia

## Abstract

**Introduction:**

Vector-borne diseases (VBDs) remain a significant global public health challenge, disproportionately affecting low- and middle-income countries. Traditional vector control methods, particularly chemical insecticides, face increasing limitations due to the rapid evolution of resistance and environmental concerns.

**Materials and methods:**

This review explores recent advancements in vector control, focusing on the integration of genomic, biological, and chemical strategies as innovative solutions to address these challenges. These methods include genomic tools such as CRISPR/Cas9-mediated systems, biological interventions like Wolbachia-based strategies and sterile insect techniques (SIT), and chemical innovations involving insecticides with novel modes of action and advanced delivery systems.

**Results:**

Genomic strategies like CRISPR/Cas9 gene drives show significant potential for precisely targeting vector reproduction and pathogen spread but face ecological and ethical hurdles to widespread use. Successful biological interventions, such as Wolbachia and SIT, have proven effective in reducing vector populations, yet they demand strong community involvement and ongoing funding for scalability. Additionally, innovative chemical solutions, including new insecticides and delivery methods, tackle resistance issues while reducing environmental harm, with techniques like microencapsulation and synergists improving sustainability.

**Discussion:**

This review highlights the importance of Integrated Vector Management (IVM) frameworks that combine genomic, biological, and chemical strategies. These integrated approaches maximize synergies while mitigating the limitations of individual methods. Key findings emphasize the potential of integrated approaches to achieve sustainable reductions in vector populations and disease transmission. However, significant challenges remain, including the need for standardized protocols, long-term effectiveness data, and considerations of ecological risks and climate change impacts.

**HIGHLIGHTS**

- This review presents a comprehensive analysis of integrating genomic, biological, and chemical strategies to combat vector-borne diseases.
- It discusses the potential of CRISPR/Cas9 gene drives, Wolbachia-based interventions, and novel insecticide technologies.
- The paper emphasizes the importance of Integrated Vector Management (IVM) in overcoming challenges such as insecticide resistance, environmental impact, and climate change, offering a holistic framework for sustainable vector control.

## Introduction

Vector-borne diseases (VBDs) represent a significant global health challenge, particularly in tropical and subtropical regions where environmental and socioeconomic conditions foster the proliferation of disease vectors. These diseases, including malaria, dengue, chikungunya, and Zika, are primarily transmitted by insect vectors such as mosquitoes, ticks, and sandflies, posing a continuous threat to millions of lives worldwide. Despite decades of research and intervention efforts, the burden of VBDs remains alarmingly high, exacerbated by emerging challenges such as climate change, urbanization, and increasing insecticide resistance. Addressing these challenges requires innovative approaches to vector control that go beyond traditional strategies and integrate advances across multiple scientific disciplines^[1-3]^.

Traditional methods of vector control, such as insecticide-treated nets (ITNs), indoor residual spraying (IRS), and environmental management, have proven effective in reducing disease incidence. However, their efficacy is increasingly undermined by the evolution of resistance in vector populations to commonly used chemical insecticides. For instance, widespread resistance to pyrethroids, the cornerstone insecticide class for ITNs and IRS, has been reported in mosquito populations across Africa, Asia, and South America. Moreover, global warming and changes in precipitation patterns are reshaping vector ecology, facilitating the expansion of vector habitats into previously non-endemic regions. Such dynamics necessitate a paradigm shift in vector control strategies, emphasizing integrated, sustainable, and innovative solutions^[4-6]^.

Recent advancements in genomic, biological, and chemical sciences have opened new avenues for vector control. Genomic technologies, including CRISPR-based gene editing and gene drive systems, offer unprecedented potential for manipulating vector populations to reduce their capacity to transmit pathogens. These tools can target specific genes involved in vector reproduction, survival, or pathogen transmission, thereby offering a sustainable and species-specific approach to control. Simultaneously, biological methods, such as the introduction of Wolbachia-infected mosquitoes, have demonstrated significant success in reducing dengue virus transmission in field trials. Wolbachia, a naturally occurring endosymbiotic bacterium, reduces the ability of mosquitoes to transmit certain viruses, providing an eco-friendly and self-sustaining control mechanism^[7-9]^.

Although CRISPR/Cas9 gene drives and Wolbachia-based interventions show great promise, they also pose specific ecological and ethical challenges. Gene drives could result in unintended gene flow to non-target species through hybridization, as observed with Anopheles mosquito complexes. Resistance development against gene drive elements has been documented in laboratory settings, potentially undermining long-term effectiveness. Additionally, ecological disruptions may arise from the suppression or elimination of vector populations, affecting food webs and biodiversity. Wolbachia dynamics could also shift over time, influencing vector competence in unpredictable ways. Therefore, thorough ecological risk assessments, post-release monitoring, and the development of reversible or localized gene drive systems are critical to mitigating these risks^[10]^.

Chemical innovations are also advancing, with the development of new classes of insecticides that are less prone to resistance and have minimal environmental impact. These include insecticides with novel modes of action, as well as the use of synergists to enhance the efficacy of existing compounds. Furthermore, the integration of chemical and biological control strategies, such as combining insecticide-treated surfaces with Wolbachia-infected mosquitoes, has shown promise in enhancing overall control efficacy while mitigating resistance risks^[11-13]^.

Given the complexity and dynamic nature of vector-borne disease transmission, an integrated approach that combines genomic, biological, and chemical strategies is imperative. This review aims to provide a comprehensive synthesis of recent advancements in vector control, highlighting innovative tools and their applications in reducing the burden of vector-borne diseases. By exploring the synergies and challenges associated with these approaches, the review underscores the potential of interdisciplinary collaboration in achieving sustainable and effective vector management. Furthermore, it seeks to identify gaps in current research and propose future directions for the development and implementation of innovative vector control strategies^[14-16]^.

## Materials and methods

In this review, a systematic and comprehensive methodology was employed to collect, analyze, and synthesize the most relevant and recent literature on innovative approaches to vector control, focusing on the integration of genomic, biological, and chemical strategies. The methods are outlined in detail below to ensure reproducibility and transparency.

### Search strategy

A comprehensive systematic literature search was performed across multiple electronic databases, including PubMed, Web of Science, Scopus, and Google Scholar, to identify relevant peer-reviewed articles, reviews, and gray literature published between January 2000 and December 2024. This timeframe was selected to encompass contemporary advancements following key technological breakthroughs, such as the introduction of CRISPR/Cas9 genome editing (first demonstrated in 2012), the resurgence of Wolbachia-based mosquito control strategies, and evolving trends in insecticide resistance management. The search strategy combined a variety of keywords, including “vector control,” “genomic strategies in vector control,” “biological control of vectors,” “chemical insecticides for disease vectors,” “integrated vector management,” “CRISPR in mosquitoes,” “Wolbachia-infected mosquitoes,” and “resistance in vector populations.” Boolean operators (AND, OR) were used to optimize search results, and specific Boolean strings were constructed, such as: (“vector control” OR “mosquito control”) AND (“genomic strategies” OR “CRISPR” OR “gene drive”) AND (“biological interventions” OR “Wolbachia” OR “sterile insect technique”) AND (“chemical insecticides” OR “resistance management”). Filters were applied to restrict the results to English-language, peer-reviewed publications, and duplicate records were excluded^[17,18]^.

### Inclusion and exclusion criteria

To maintain the quality and relevance of the selected studies, the following criteria were applied. Inclusion criteria required articles to be published in peer-reviewed journals, focus on genomic, biological, or chemical approaches to vector control, and include reviews, original research, or meta-analyses addressing innovative or integrated strategies. Papers highlighting field trials, laboratory studies, or computational models were also included. Exclusion criteria removed articles unrelated to vector control or focused on non-medical pests, studies with insufficient methodological details, and publications older than 2000 unless considered seminal works. The initial search identified 2340 articles, and after removing duplicates, 1780 articles remained. Title and abstract screening yielded 256 articles for full-text review, of which 172 were included in the final analysis^[19,20]^.

### Data extraction

A standardized data extraction form was used to systematically collect detailed information from the selected studies, including study design (laboratory, field, or computational), target vector species and geographic region, type of control strategy (genomic, biological, or chemical), outcome measures (such as vector population reduction, resistance mitigation, or disease incidence), and key findings and conclusions. At each stage of the review process (title/abstract screening and full-text review), two independent reviewers conducted the screening and data extraction to minimize bias. Discrepancies between reviewers were resolved through discussion, and, if consensus could not be reached, a third senior reviewer adjudicated the decision. Inter-rater reliability was assessed using Cohen’s Kappa coefficient, which yielded a value of 0.82, indicating substantial agreement between reviewers^[21,22]^.

### Classification of strategies

The identified vector control strategies were categorized into three main domains. These included gene editing techniques like CRISPR/Cas9, gene drive systems targeting vector reproduction and pathogen transmission, and transcriptomic and proteomic studies to inform target identification (Genomic Approaches). Strategies included Wolbachia-infected mosquito programs, the use of natural predators and parasites for vector suppression, and the integration of biological strategies with community-based interventions (Biological Approaches). These involved the development of insecticides with novel modes of action, the synergistic use of chemical compounds with biological agents, and innovations in delivery mechanisms such as microencapsulation and spatial repellents (Chemical Approaches)^[8,9,13]^.

### Critical appraisal and quality assessment

Each study was critically appraised using validated quality assessment tools, such as the Joanna Briggs Institute Critical Appraisal Tools for reviews and experimental studies. Studies were scored on aspects such as methodological rigor, reproducibility, and relevance to current vector control challenges. Only studies scoring above a pre-defined threshold (≥75%) were included in the synthesis^[23,24]^.

### Data synthesis and analysis

A narrative synthesis approach was employed to integrate findings across the three domains. Key themes included synergies between genomic, biological, and chemical approaches, regional variations in strategy implementation and effectiveness, and gaps in current knowledge requiring further research. Quantitative data were summarized in tables and figures to facilitate comparisons. Due to heterogeneity in study designs and outcome measures, meta-analytic techniques were not applied^[20,25]^.

### Ethical considerations

**Figure 1.**
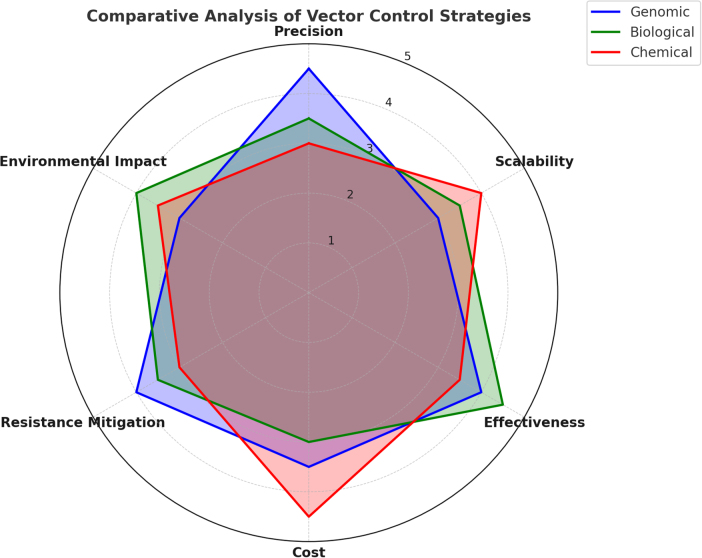
Comparative performance analysis of genomic, biological, and chemical vector-control strategies.

This review did not involve primary data collection or human/animal subjects. All included studies were assumed to have adhered to ethical guidelines, as summarized in Table 1 and illustrated in Fig. 1^[26,27]^.

**Table 1:** Summary of Findings on Genetic Strategies, Biological Interventions, and Chemical Innovations in Vector Control

Field	Strategy/Method	Key Findings	Challenges and Limitations	Future Opportunities
GeneticStrategies	CRISPR/Cas9 and Gene Drive	Genetic technologies, especially gene drives, have successfully reduced mosquito populations in laboratory settings.	Environmental concerns, ethical issues, and potential resistance in mosquitoes.	Development of reversible and localized gene drives to minimize risks and improve public acceptance.
	Transcriptomic and Proteomic Analysis	Identified key genes in insecticide resistance and pathogen transmission, providing new targets to disrupt disease transmission.	Scalability issues and application in natural environments.	Identification and targeting of specific genes to combat insecticide resistance.
BiologicalInterventions	Wolbachia	Use of Wolbachia-infected mosquitoes significantly reduced the prevalence of diseases like dengue and Zika.	Dependence on environmental conditions and the need for repeated mosquito releases.	Combining Wolbachia with other methods, like gene drives or SIT, to improve effectiveness.
	Sterile Insect Technique (SIT)	The SIT approach has shown positive results in controlling mosquito populations, particularly in specific regions.	High implementation costs and logistical challenges, especially in low-resource countries.	Optimizing and integrating SIT with genetic modifications to enhance efficiency.
Chemical Innovations	Novel Insecticides and Synergists	Development of insecticides with novel modes of action and synergists, such as piperonyl butoxide (PBO), has mitigated resistance issues.	Continuous application requirements and the risk of increased resistance.	Combining novel insecticides with biological and genetic methods to reduce reliance on chemicals.
	Innovative Delivery Systems	Innovations like microencapsulation and spatial repellents have improved insecticide efficiency and safety.	Environmental risks and the potential for resistance to insecticides.	Using innovative delivery systems in combination with other strategies to enhance control measures.
Integration and Synergies	Combining Strategies	Integration of genetic, biological, and chemical methods has led to better results in reducing mosquito populations and disease transmission.	Challenges in coordination and implementation across different environments.	Development of Integrated Vector Management (IVM) models for effective strategy integration.

## Results

This review synthesizes recent advancements in vector control strategies, focusing on the integration of genomic, biological, and chemical approaches. The findings are categorized into three primary domains: genomic strategies, biological interventions, and chemical innovations, highlighting key outcomes, challenges, and potential synergies^[28,29]^.

Genomic tools, particularly those leveraging CRISPR/Cas9 technology, have revolutionized vector control research. Several studies have demonstrated the potential of gene drive systems to suppress vector populations. Gene drives targeting mosquito reproductive genes, such as doublesex, have shown success in laboratory trials, with significant reductions in fertility and population size within a few generations. Field trials, while limited, have indicated promising results, particularly in isolated ecosystems where ecological risks are more manageable. Additionally, transcriptomic and proteomic analyses have provided new insights into vector biology and pathogen-vector interactions. Studies have identified critical genes responsible for insecticide resistance and pathogen transmission, offering novel targets for genetic disruption. For example, mutations in the voltage-gated sodium channel (VGSC) gene associated with pyrethroid resistance have been targeted using RNA interference (RNAi) and CRISPR, effectively restoring insecticide susceptibility in lab-based experiments. However, challenges persist in scaling these technologies for field use. Ecological risks, ethical concerns, and public acceptance remain significant barriers. Furthermore, the potential for resistance to gene drives underscores the need for integrated strategies that combine genomic tools with other approaches^[8,30,31]^.

The use of Wolbachia-infected mosquitoes has emerged as a groundbreaking biological intervention. Wolbachia, a symbiotic bacterium, reduces the capacity of mosquitoes to transmit arboviruses, including dengue, Zika, and chikungunya. Large-scale field trials, such as those conducted in Indonesia and Australia, have reported reductions of up to 70% in dengue incidence in areas where Wolbachia-infected mosquitoes were released. Other biological approaches, such as the use of sterile insect techniques (SIT), have also shown promise. SIT involves irradiating male mosquitoes to induce sterility, and its deployment in combination with Wolbachia has been tested to enhance efficacy. These approaches have proven particularly effective in controlling isolated vector populations. Natural predators and parasites, including fish species such as Gambusia and fungal pathogens like Beauveria bassiana, continue to play a role in integrated pest management. While these methods are environmentally friendly, their scalability and specificity remain limited compared to other interventions. The success of biological interventions is highly dependent on local ecological and social contexts. Community engagement and sustained funding are critical for the long-term implementation of such strategies^[32-34]^.

Chemical control methods remain a cornerstone of vector management, but recent innovations are addressing limitations associated with traditional insecticides. New classes of insecticides with novel modes of action, such as metabolic disruptors and juvenile hormone analogs, have been developed to overcome resistance. For instance, pyriproxyfen, a juvenile hormone analog, has shown efficacy in disrupting mosquito development at extremely low doses. The use of synergists, such as piperonyl butoxide (PBO), has been integrated into insecticide formulations to restore susceptibility in resistant populations. Studies have demonstrated that PBO-treated nets significantly reduce malaria transmission in regions with pyrethroid-resistant vectors. Innovative delivery systems, such as microencapsulation and spatial repellents, have enhanced the efficiency and environmental safety of chemical interventions. Microencapsulation allows for the slow and controlled release of insecticides, reducing the need for frequent applications and minimizing non-target effects. Spatial repellents, including volatile pyrethroids, have shown effectiveness in preventing mosquito entry into human dwellings. However, reliance on chemical methods raises concerns about environmental toxicity and the emergence of resistance. Integrated approaches that combine chemical control with biological and genomic strategies are necessary to mitigate these risks^[5,35,36]^.

While PBO-treated nets have significantly improved malaria control outcomes in regions with pyrethroid-resistant vectors, their long-term effectiveness remains uncertain. The potential evolution of new resistance mechanisms and vector behavioral changes necessitates longitudinal studies across diverse ecological and socio-economic settings. Furthermore, the successful implementation of integrated vector control strategies that combine genomic, biological, and chemical methods requires careful operational planning, adaptive management, community engagement, and investment in local capacity-building initiatives. Coordination between public health authorities, research institutions, and communities will be critical to ensuring the sustainability and scalability of integrated approaches^[37-39]^.

The integration of genomic, biological, and chemical strategies offers significant potential to enhance the sustainability and effectiveness of vector control programs. For example, combining Wolbachia-infected mosquitoes with targeted insecticide use has shown synergistic effects in reducing both vector populations and transmission rates. Similarly, gene-editing tools can be used to enhance the efficacy of sterile insect techniques by improving the fitness of released males. Models of Integrated Vector Management (IVM) that incorporate community-based approaches have been particularly successful in addressing region-specific challenges. Involving local populations in the design and implementation of control programs ensures greater acceptance and long-term sustainability^[40,41]^.

To address the cost-efficiency challenges associated with advanced vector control strategies, integrated approaches that combine genomic, biological, and chemical tools are essential. The development of Integrated Vector Management (IVM) frameworks, technological innovations to optimize intervention methods, and strong community engagement are critical steps toward improving the economic feasibility and scalability of these strategies, particularly in low-resource settings. Regional and international collaboration can play a pivotal role in addressing financial and operational challenges associated with Integrated Vector Management (IVM). By fostering partnerships for resource sharing, capacity building, and research innovation, countries can collectively bridge financial gaps, harmonize policies, and enhance the sustainability and scalability of vector control programs. Such collective action is essential for transforming IVM frameworks from conceptual models into operational realities, particularly in resource-constrained settings. Although integrated vector management strategies offer promising results, their implementation in resource-limited settings faces significant barriers. Successful programs, such as the Wolbachia deployments in Indonesia and integrated initiatives in Tanzania, highlight the importance of phased rollouts, community engagement, international partnerships, and strengthening local capacity to overcome these challenges^[42-44]^.

Despite substantial progress, several gaps remain in the field of vector control. Limited understanding of the ecological impacts of genomic and biological interventions poses a challenge to their widespread implementation. Moreover, insufficient infrastructure in low-income countries limits access to advanced tools and technologies. The lack of comprehensive data on the long-term effectiveness and cost-efficiency of integrated approaches highlights the need for further research. Establishing standardized protocols for field trials and data collection will be critical in addressing these gaps^[45,46]^.

### Summary of key findings

Genomic strategies, such as gene drives, offer transformative potential but face ecological and ethical challenges. Biological methods, particularly Wolbachia-infected mosquitoes, have achieved significant success in reducing disease transmission. Chemical innovations, including new insecticides and delivery systems, continue to address resistance and environmental concerns. Integrated approaches that leverage synergies among these strategies are essential for sustainable vector management^[46,47]^.

## Discussion

The field of vector control has witnessed significant advancements over recent decades, driven by the urgent need to address the global burden of vector-borne diseases (VBDs). This discussion evaluates the findings presented in this review, explores their implications, and considers the challenges and future directions of integrating genomic, biological, and chemical strategies into comprehensive vector management programs^[48-50]^.

Genomic tools, particularly CRISPR/Cas9 and gene drive systems, have opened transformative possibilities for vector control. These approaches target vector reproduction and pathogen transmission with unparalleled precision, offering long-term, self-sustaining solutions. For example, gene drive systems targeting the doublesex gene in mosquitoes have demonstrated the potential to collapse populations in controlled environments. However, their deployment in natural settings remains contentious due to ecological and ethical concerns. Critics argue that gene drives may inadvertently affect non-target species or disrupt ecosystems. Additionally, the potential for resistance evolution within vector populations poses a significant challenge. As highlighted in this review, combining gene drives with other strategies, such as Wolbachia-based interventions or chemical controls, could mitigate these risks while enhancing efficacy. Future research should focus on developing reversible or localized gene drives to limit unintended consequences and improve public acceptance^[51-53]^.

While gene drive systems hold transformative potential for vector control, they also raise substantial ecological and ethical concerns. Unintended gene flow to non-target species through hybridization could disrupt local biodiversity and ecosystems. Moreover, the suppression of mosquito populations could alter food webs, potentially impacting predators and ecosystem balance. Resistance to gene drives may emerge through natural selection, thereby reducing their long-term efficacy. From an ethical standpoint, the release of gene drive organisms requires careful consideration of community consent, cross-border regulatory frameworks, and the possibility of irreversible ecological changes. Thorough ecological risk assessments, development of confinable or reversible gene drive technologies, and broad stakeholder engagement are essential to responsibly advance the application of genomic tools in vector control. While laboratory studies provide valuable proof-of-concept data for innovative vector control technologies, translating these results into natural environments presents significant challenges. Environmental heterogeneity, complex ecological interactions, vector behavioral adaptations, and evolutionary pressures can profoundly affect intervention outcomes. For example, gene drive constructs that successfully collapse mosquito populations in the laboratory may encounter resistance evolution or unexpected ecological barriers in the field. Similarly, Wolbachia-infected mosquito programs have demonstrated variable persistence across different climatic and ecological contexts. The sterile insect technique (SIT) also faces challenges in maintaining male mosquito competitiveness under natural conditions. Therefore, robust, long-term field evaluations, adaptive management strategies, and ecological modeling are critical for validating the real-world applicability and sustainability of these technologies^[54-57]^.

Biological interventions, such as Wolbachia-based strategies and the sterile insect technique (SIT), have emerged as environmentally sustainable alternatives to chemical insecticides. Field trials in countries like Indonesia and Australia have shown remarkable success in reducing the incidence of diseases like dengue. The compatibility of Wolbachia with existing vector control programs further underscores its potential as a scalable solution. However, the effectiveness of these strategies depends heavily on the biological and ecological characteristics of the target vector species and local environments. For instance, the introduction of Wolbachia-infected mosquitoes may be less effective in regions with high seasonal variations or where non-target vector species contribute to disease transmission. Additionally, maintaining the stability of Wolbachia infections in mosquito populations over time requires careful monitoring and repeated releases.

While innovative vector control strategies, such as Wolbachia-based interventions and sterile insect technique (SIT), have shown promise in controlled trials, their scalability and feasibility remain significant challenges in low-resource settings. For instance, programs in Brazil revealed that maintaining high Wolbachia infection rates required repeated releases and ongoing community engagement, contributing to high operational costs. Similarly, SIT initiatives in Africa have encountered difficulties related to the establishment of large-scale rearing facilities, sex-sorting technology, and consistent field releases. Political, infrastructural, and logistical barriers further complicate large-scale deployment in many endemic regions. Addressing these challenges requires context-specific adaptation, international support, and investment in local capacity-building initiatives to ensure the sustainability of these approaches^[58-60]^.

The integration of SIT with genetic modifications to improve the fitness of sterile males represents a promising avenue for enhancing the efficacy of biological methods. However, cost and logistical constraints remain significant barriers to the widespread adoption of SIT, particularly in low-resource settings^[61-63]^.

Despite the challenges posed by insecticide resistance, chemical control remains a cornerstone of vector management. Recent innovations, such as the development of insecticides with novel modes of action and synergists like piperonyl butoxide, have revitalized the field. These advancements address resistance issues while minimizing environmental impact. Additionally, innovations in delivery mechanisms, such as microencapsulation, provide a more targeted and sustainable approach to chemical interventions.

However, chemical methods are inherently limited by their dependency on continuous application and the risk of resistance development. This review highlights the importance of integrating chemical strategies with genomic and biological methods to reduce reliance on insecticides. For example, combining Wolbachia-infected mosquitoes with spatial repellents or targeted insecticide applications could enhance overall program effectiveness while minimizing ecological harm^[5,64,65]^.

The integration of genomic, biological, and chemical strategies offers a holistic approach to vector control. Such integration not only leverages the strengths of each method but also mitigates their respective limitations. For example, the simultaneous use of gene drives and Wolbachia-infected mosquitoes could accelerate population suppression while reducing the risk of resistance. Similarly, combining chemical insecticides with biological control methods, such as predators or pathogens, could provide immediate and sustained reductions in vector populations. The implementation of Integrated Vector Management (IVM) frameworks is critical for achieving these synergies. IVM emphasizes a multi-sectoral approach that incorporates ecological, social, and economic considerations. Community engagement is particularly crucial, as the success of interventions like Wolbachia releases and indoor residual spraying depends on public acceptance and participation. Governments and international organizations must invest in capacity-building and infrastructure development to facilitate the adoption of IVM in low-resource settings^[46,66,67]^.

The deployment of advanced technologies, like gene drives and Wolbachia-infected mosquitoes, necessitates careful navigation of political and regulatory landscapes. International agreements, such as the Cartagena Protocol, emphasize the need for precautionary measures, while national policies may vary widely. Balancing public health imperatives with ecological and ethical concerns requires transparent governance, phased testing, community engagement, and robust international coordination^[68-70]^.

The use of Wolbachia-infected mosquitoes represents a promising biological intervention, but its effectiveness and sustainability are influenced by several ecological and operational factors. Environmental variables, such as high ambient temperatures, can reduce Wolbachia density and impair its ability to block pathogen transmission. Strain selection is critical, as some strains, like wMel, are more sensitive to heat stress, whereas wAlbB exhibits greater thermal tolerance. Additionally, evolutionary dynamics within mosquito populations or the Wolbachia bacterium itself may influence infection stability over time. Ensuring long-term sustainability requires robust, continuous entomological surveillance to detect changes in Wolbachia prevalence and mosquito population dynamics. In some settings, periodic supplementary releases may be necessary to maintain effective coverage, highlighting the need for flexible, adaptive management strategies tailored to local ecological contexts^[71-73]^.

The interaction between Wolbachia-based interventions and chemical control strategies presents opportunities for synergistic or conflicting outcomes. Strategic, localized insecticide applications can suppress wild-type mosquito populations while maintaining Wolbachia prevalence, enhancing overall vector control effectiveness. However, indiscriminate insecticide use risks undermining Wolbachia establishment, necessitating careful program design. In parallel, the emergence of resistance to next-generation insecticides poses a growing concern. Although these compounds (e.g., neonicotinoids, pyriproxyfen) target novel biochemical pathways, mosquitoes may develop resistance through metabolic mechanisms, such as overexpression of detoxifying enzymes, or through target-site modifications. Furthermore, cross-resistance between traditional and new insecticides could occur if resistance mechanisms overlap. Continuous surveillance, strategic insecticide rotation, and integration with biological methods are essential to prolong the operational lifespan of chemical interventions and mitigate resistance risks^[74-78]^.

Despite substantial progress, several challenges persist in the field of vector control. The ecological and ethical implications of genomic interventions remain poorly understood, underscoring the need for robust risk assessments and regulatory frameworks. Additionally, limited access to advanced technologies in low- and middle-income countries exacerbates disparities in the global fight against VBDs. Another significant gap is the lack of long-term data on the effectiveness and cost-efficiency of integrated strategies. Most studies focus on short-term outcomes, leaving questions about sustainability unanswered. Furthermore, the influence of climate change on vector ecology and the effectiveness of control strategies is an area that requires urgent attention^[79-81]^.

Predictive models integrating climate change variables are increasingly essential tools for vector control planning. Dynamic transmission and ecological niche models can forecast shifts in vector distribution, allowing for proactive, geographically targeted interventions. Climate-resilient vector control strategies must incorporate such forecasting tools to remain effective under evolving environmental conditions. Vector control strategies must be adapted to ecological and social contexts. In tropical regions with perennial vector presence, interventions often focus on continuous suppression (e.g., Wolbachia deployments, larval source management). In temperate regions, seasonal interventions targeting peak transmission periods may be more effective. Similarly, urban settings may prioritize indoor residual spraying and community education, while rural areas may benefit from integrated approaches combining environmental management with biological control. Recognizing these differences is essential for tailoring Integrated Vector Management (IVM) frameworks to local needs^[82-85]^.

The findings of this review have important implications for public health policy and research priorities. Policymakers should prioritize funding for interdisciplinary research that bridges genomic, biological, and chemical sciences. Establishing global collaborations to standardize protocols for field trials and data collection will enhance the comparability of results and facilitate the scaling of successful interventions.

Climate change represents a significant emerging challenge for vector control strategies. Changes in temperature, precipitation, and humidity patterns are altering the geographic distribution and seasonal dynamics of key vectors, expanding their range into previously non-endemic regions. For example, *Aedes* mosquitoes are now reported in parts of Europe and North America where they were previously absent. Climate variability can also increase vector breeding rates and pathogen transmission potential. Consequently, the long-term effectiveness of current control strategies may be compromised unless they are adapted to account for climate-induced changes. Integrating climate modeling, real-time surveillance, and flexible intervention approaches will be essential for sustaining the effectiveness of vector control programs in a rapidly changing environment^[86-90]^.

Future research should focus on: Developing localized gene drive systems with reversible mechanisms, enhancing the cost-efficiency of biological methods like Wolbachia and SIT, innovating environmentally safe and resistance-proof insecticides, and exploring the impact of climate change on the efficacy of vector control strategies. Finally, capacity-building initiatives and public engagement campaigns are essential to ensure the successful implementation of integrated vector management programs^[91-93]^.

## Conclusion

This discussion underscores the transformative potential of integrating genomic, biological, and chemical strategies in vector control. While significant challenges remain, the synergies offered by these approaches provide a pathway toward sustainable and effective management of vector-borne diseases. By addressing current gaps and fostering interdisciplinary collaboration, the global health community can achieve a meaningful reduction in the burden of these diseases, improving public health outcomes worldwide.

## Data Availability

All data obtained from this study are included in the text of the article.

## Research registration unique identifying number (UIN)

This is a review study and does not require an ethical code.
